# *METTL1* gene polymorphisms synergistically confer hepatoblastoma susceptibility

**DOI:** 10.1007/s12672-022-00545-7

**Published:** 2022-08-20

**Authors:** Lili Ge, Jinhong Zhu, Jiabin Liu, Li Li, Jiao Zhang, Jiwen Cheng, Yong Li, Zhonghua Yang, Suhong Li, Jing He, Xianwei Zhang

**Affiliations:** 1 Henan Provincial Key Laboratory of Children’s Genetics and Metabolic Diseases, Children’s Hospital Affiliated to Zhengzhou University, Henan Children’s Hospital, Zhengzhou Children’s Hospital, Zhengzhou, 450018 Henan China; 2 Department of Clinical Laboratory, Biobank, Harbin Medical University Cancer Hospital, Harbin, 150040 Heilongjiang China; 3 Department of Pediatric Surgery, Guangzhou Institute of Pediatrics, Guangdong Provincial Key Laboratory of Research in Structural Birth Defect Disease, Guangzhou Women and Children’s Medical Center, Guangzhou Medical University, 9 Jinsui Road, Guangzhou, 510623 Guangdong China; 4 Kunming Key Laboratory of Children Infection and Immunity, Yunnan Key Laboratory of Children’s Major Disease Research, Yunnan Institute of Pediatrics Research, Yunnan Medical Center for Pediatric Diseases, Kunming Children’s Hospital, Kunming, 650228 Yunnan China; 5 Department of Pediatric Surgery, The First Affiliated Hospital of Zhengzhou University, Zhengzhou, 450052 Henan China; 6 Department of Pediatric Surgery, The Second Affiliated Hospital of Xi’an Jiaotong University, Xi’an, 710004 Shaanxi China; 7 Department of Pediatric Surgery, Hunan Children’s Hospital, Changsha, 410004 Hunan China; 8 Department of Pediatric Surgery, Shengjing Hospital of China Medical University, Shenyang, 110004 Liaoning China; 9 Department of Pathology, Children Hospital and Women Health Center of Shanxi, Taiyuan, 030013 Shannxi China; 10 Department of Pediatric Oncologic Surgery, Children’s Hospital Affiliated to Zhengzhou University, Henan Children’s Hospital, Zhengzhou Children’s Hospital, 33 Longhu Waihuan East Road, Zhengzhou, 450018 Henan China

**Keywords:** Hepatoblastoma, Susceptibility, *METTL1*, Polymorphism, m7G modification

## Abstract

**Introduction:**

Hepatoblastoma is a rare but devastating pediatric liver malignancy. Overexpressed methyltransferase-like 1 (METTL1) is a methyltransferase that catalyzes essential N7-methylguanosine (m7G) modification of eukaryotic mRNA. Accumulating evidence has revealed the oncogenic potential of METTL1. However, whether *METTL1* gene polymorphisms confer susceptibility to hepatoblastoma has not been reported. This study aimed to identify causal relationships between genetic variants of this gene and susceptibility to hepatoblastoma.

**Materials and methods:**

Using the TaqMan assay, we genotyped three *METTL1* polymorphisms (rs2291617 G > T, rs10877013 T > C, rs10877012 T > G) in germline DNA samples from 1759 Chinese children of Han ethnicity (313 cases vs. 1446 controls).

**Results:**

None of these polymorphisms were associated with hepatoblastoma risk. However, combination analysis showed that children with 1 to 3 risk genotypes were associated with increased hepatoblastoma risk (adjusted odds ratio = 1.47, 95% confidence interval  1.07–2.02; *P* = 0.018). Stratified analyses revealed significant effects of combined polymorphisms mainly among young children (< 17 months of age), boys, and those with advanced hepatoblastoma.

**Conclusion:**

We identified some potential functional *METTL1* gene polymorphisms that work together to increase the risk of hepatoblastoma among Chinese Han children; single polymorphism showed only weak effects. These *METTL1* polymorphisms may be promising biomarkers for screening high-risk individuals for hepatoblastoma. These findings are inspiring and deserve to be validated among individuals of different ethnicities.

**Graphical Abstract:**

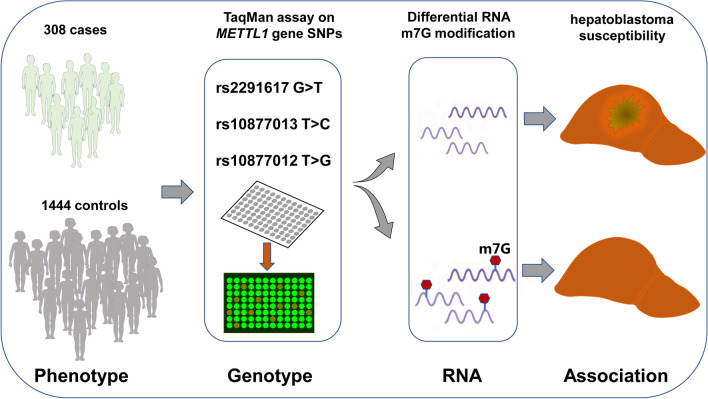

**Supplementary Information:**

The online version contains supplementary material available at 10.1007/s12672-022-00545-7.

## Introduction

Hepatoblastoma is the most frequently diagnosed malignant liver tumor, accounting for 1% of all pediatric tumors [1]. Pediatric hepatoblastoma mainly affects children aged between 6 months and 3 years, and the annual incidence is approximately 0.5–1.5 per million children [1, 2]. The five-year overall survival has been raised from 30% to approximately 80% due to multimodality therapy, including cisplatin- and anthracycline-based chemotherapy regimens, and advanced surgical techniques [3, 4]. Unfortunately, high-risk patients have poor prognoses, including those with large tumor masses, older children (≥ 8 years old) and those with tumor metastasis or low concentrations of AFP (α fetoprotein ≤ 100 ng/mL) at diagnosis, and there is considerable room for improvement regarding outcomes [5]. As a result of extremely low incidence, the etiology of hepatoblastoma has yet to be fully elucidated [1]. Prematurity, low birth weight, and parental smoking may increase the risk for hepatoblastoma [3, 6].

In addition to environmental causes, genetic alterations also contribute to the development of hepatoblastoma. For instance, although the majority of hepatoblastoma cases are rare, evidence shows that some genetic cancer syndromes, including Beckwith–Wiedemann syndrome (BWS) and familial adenomatous polyposis (FAP), predispose children to hepatoblastoma [3]. Currently, there are no genome-wide association studies (GWASs) examining hepatoblastoma. Several groups, including ours, have demonstrated that some genetic variants in the *LINC00673*, *NRAS*, *KRAS*, *TP53*, *HMGA2*, *miR-34b/c*, *YTHDF1*, and *WTAP* genes were associated with hepatoblastoma susceptibility [7–12]. However, given the limited number of susceptibility genes and small sample size, many more functional genetic variants in essential genes should be investigated in large cohorts.

Methyltransferase-like 1 (METTL1) was initially identified to catalyze the modification of N7-methylguanosine (m7G) at the 5’ cap of eukaryotic mRNA [13]. This cap modification plays a pivotal role in stabilizing transcripts and regulating transcription elongation, pre-mRNA splicing, polyadenylation, nuclear export, and translation [14]. Recently, in addition to its location in the cap structure, METTL1-mediated m7G was also found internally within tRNA, rRNA, and mRNA [14]. *METTL1* is mapped to chromosome 12 (12 q13-14), a region known to be frequently amplified in cancers [15]. Some groups found that METTL1 is tumor-promoting in colon cancer, hepatocellular carcinoma, and head and neck squamous cell carcinoma [15–19]; additionally, opposite findings have also been reported [20, 21]. To date, the impacts of METTL1 in hepatoblastoma remain unknown. The current case-control study aimed to investigate the association of three single-nucleotide polymorphisms (SNPs) in the *METTL1* gene with hepatoblastoma susceptibility in 313 pediatric patients and 1446 healthy controls.

## Materials and methods

### Study population

Details regarding subject recruitment were described in previous publications [11, 22]. We enrolled 313 children with hepatoblastoma and 1446 controls of Chinese Han ethnicity from seven independent hospitals located in different cities (i.e., Guangzhou, Kunming, Xi’an, Zhengzhou, Changsha, Taiyuan, and Shenyang), thus involving most of the geographic regions from south to northeast China.

The inclusion and exclusion criteria for hepatoblastoma patients were as follows: (1) Han Chinese ethnicity, (2) newly diagnosed hepatoblastoma with histopathological confirmation, (3) no familial disorder or family history of cancer, and (4) aged 14 years or younger. Patients who received medical intervention or failed to provide signed informed consent were excluded. The characteristics of the study cohort, including age, sex and clinical stages of hepatoblastoma, are listed in the supplemental materials (Additional file 1: Table S1). We staged the patients in accordance with the PRETEXT classification [23]. Control subjects were healthy children visiting the same hospitals for the same routine health examinations as the cases. They also had no family history of cancer or inherited diseases. The parents or guardians of each subject provided signed informed consent. The study protocol acquired approval from the institutional review board of Guangzhou Women and Children’s Medical Center (No: 202016601).

### Selection and genotyping of SNPs

We selected potential functional SNPs in the *METTL1* gene following standard criteria [24, 25]. We chose SNPs with a minor allele frequency of 5% or higher in Chinese Han subjects that were potentially functional as predicted by SNPinfo online software (https://snpinfo.niehs.nih.gov/snpinfo/snpfunc.html). The SNP rs2291617 G > T is located in the 5′ near region, rs10877013 T > C is located in the intron region, and rs10877012 T > G is located in the 3′ near region. All of these three polymorphisms can affect transcription factor-binding site (TFBS) activity. Genomic DNA was collected from the participants’ peripheral blood samples using the Tiangen Blood DNA Extraction kit (Tiangen Biotechnology, Beijing, China). Genotypes of samples were examined using a TaqMan platform (Applied Biosystems, Foster City, CA) with the inclusion of both negative and positive control samples in each 384-well plate. The experiments were conducted by blinded laboratory workers. To ensure the accuracy of genotyping, a proportion of the randomly selected sample was repeatedly tested. For the same samples, we obtained concordance rates of 100% in duplicate tests.

### Statistical analysis

Significant differences in clinical variables were determined between the case and control groups with a t-test for continuous variables or *χ*^2^ test for categorical variables. Hardy-Weinberg equilibrium (HWE) was evaluated by comparing the theoretical distribution of genotypes with the observed genotypes in the controls with a goodness-of-fit *χ*^2^ test. Finally, the robustness of the association of SNPs with hepatoblastoma risk was estimated using unconditional logistic regression analysis. Odds ratios (ORs) and 95% confidence intervals (CIs) were calculated for the association of SNPs with hepatoblastoma susceptibility. Stratified analyses were performed by age, sex, and clinical stage. Haplotype analysis was also performed [22]. A two-sided *P* < 0.05 was accepted as statistically significant. SAS v10.0 (SAS Institute Inc., Cary, NC) was adopted to implement all analyses.

## Results

### **Association of hepatoblastoma risk with*METTL1*SNPs**

In this study, we genotyped three *METTL1* SNPs (rs2291617 G > T, rs10877013 T > C, rs10877012 T > G) in 1759 samples (313 cases vs. 1446 controls) and successfully obtained genotype results for 308 cases and 1444 controls. The SNP genotypes of hepatoblastoma patients and controls are displayed in Table 1. As shown, the genotype frequencies of the three SNPs were consistent with the HWE genetic balance in control subjects (HWE = 0.407 for rs2291617 G > T, HWE = 0.632 for rs10877013 T > C, HWE = 0.672 for rs10877012 T > G). Logistic regression analyses revealed that none of the single SNPs were significantly associated with hepatoblastoma susceptibility. Furthermore, the combined analyses of risk genotypes of these SNPs revealed that children with 1 to 3 risk genotypes are at a significantly elevated risk of developing hepatoblastoma compared to noncarriers (adjusted OR = 1.47, 95% CI  1.07–2.02, *P* = 0.018).

**Table 1:** The relationship between *METTL1* gene polymorphisms and hepatoblastoma risk

Genotype	Cases (N = 308)	Controls (N = 1444)	*P* ^a^	Crude OR (95% CI)	*P*	Adjusted OR (95% CI) ^b^	*P* ^b^
rs2291617 G > T (HWE = 0.407)							
GG	122 (39.61)	589 (40.79)		1.00		1.00	
GT	133 (43.18)	655 (45.36)		0.98 (0.75–1.28)	0.885	0.98 (0.75–1.28)	0.860
TT	53 (17.21)	200 (13.85)		1.28 (0.89–1.83)	0.180	1.28 (0.89–1.83)	0.184
Additive			0.298	1.10 (0.92–1.31)	0.298	1.10 (0.92–1.31)	0.308
Dominant	186 (60.39)	855 (59.21)	0.702	1.05 (0.82–1.35)	0.702	1.05 (0.81–1.35)	0.725
Recessive	255 (82.79)	1244 (86.15)	0.128	1.29 (0.93–1.80)	0.129	1.29 (0.93–1.80)	0.129
rs10877013 T > C (HWE = 0.632)							
TT	136 (44.16)	618 (42.80)		1.00		1.00	
TC	130 (42.21)	647 (44.81)		0.91 (0.70–1.19)	0.500	0.91 (0.70–1.19)	0.489
CC	42 (13.64)	179 (12.40)		1.07 (0.73–1.57)	0.743	1.07 (0.73–1.56)	0.749
Additive			0.978	1.00 (0.83–1.20)	0.978	1.00 (0.83–1.19)	0.968
Dominant	172 (55.84)	826 (57.20)	0.662	0.95 (0.74–1.21)	0.662	0.94 (0.74–1.21)	0.650
Recessive	266 (86.36)	1265 (87.60)	0.552	1.12 (0.78–1.60)	0.552	1.12 (0.78–1.60)	0.553
rs10877012 T > G (HWE = 0.672)							
TT	130 (42.21)	581 (40.24)		1.00		1.00	
TG	126 (40.91)	664 (45.98)		0.85 (0.65–1.11)	0.230	0.85 (0.65–1.11)	0.223
GG	52 (16.88)	199 (13.78)		1.17 (0.82–1.67)	0.398	1.17 (0.81–1.67)	0.404
Additive			0.795	1.02 (0.86–1.22)	0.795	1.02 (0.86–1.22)	0.808
Dominant	178 (57.79)	863 (59.76)	0.522	0.92 (0.72–1.18)	0.522	0.92 (0.72–1.18)	0.509
Recessive	256 (83.12)	1245 (86.22)	0.158	1.27 (0.91–1.77)	0.159	1.27 (0.91–1.77)	0.160
Combined effect of risk genotypes ^c^							
0	248 (80.52)	1240 (85.87)		1.00		1.00	
1–3	60 (19.48)	204 (14.13)	0.017	**1.47 (1.07–2.02)**	**0.018**	**1.47 (1.07–2.02)**	**0.018**

The results were in bold if the 95% CI excluded 1 or *P* value less than 0.05

*OR* odds ratio; *CI* confidence interval; *HWE* Hardy-Weinberg equilibrium.

^a^*χ*^2^ test for genotype distributions between hepatoblastoma patients and cancer-free controls.

^b^Adjusted for age and gender.

^c^Risk genotypes were carriers with rs2291617 TT, rs10877013 CC and rs10877012 GG

### Stratified analyses

To more precisely evaluate the effects of SNPs on different populations of children, we stratified participants by age, sex, and clinical stage (Table 2). Stratified analyses indicated that significant results found for children carrying 1 to 3 minor alleles were mainly observed among children under 17 months of age (adjusted OR = 1.59, 95% CI  103–2.45, *P* = 0.037), boys (adjusted OR = 1.54, 95% CI  1.03–3.30, *P* = 0.036), and those with stage III or IV hepatoblastoma (adjusted OR = 1.75, 95% CI  1.04–2.95, *P* = 0.034). Overall, our findings highlight the additive effects of multiple weak penetrating SNPs and the importance of narrowing the susceptible population.

**Table 2:** Stratification analysis for the association between *METTL1* gene genotypes and hepatoblastoma risk

Variables	rs2291617 (case/control)		AOR (95% CI) ^a^	*P* ^a^	rs10877012 (case/control)		AOR (95% CI) ^a^	*P* ^a^	Risk genotypes (case/control)		AOR (95% CI) ^a^	*P* ^a^
	GG/GT	TT			TT/TG	GG			0	1–3		
Age, month												
< 17	134/553	30/88	1.38 (0.87–2.17)	0.172	137/551	27/90	1.18 (0.74–1.89)	0.487	129/549	35/92	**1.59 (1.03–2.45)**	**0.037**
≥ 17	121/691	23/112	1.17 (0.72–1.91)	0.520	119/694	25/109	1.34 (0.83–2.16)	0.228	119/691	25/112	1.30 (0.81–2.09)	0.281
Gender												
Female	109/509	17/76	1.07 (0.61–1.88)	0.824	108/520	18/75	1.16 (0.66–2.01)	0.608	105/518	21/77	1.35 (0.80–2.28)	0.268
Male	146/725	36/124	1.43 (0.95–2.17)	0.087	148/725	34/124	1.34 (0.88–2.03)	0.175	143/722	39/127	**1.54 (1.03–3.30)**	**0.036**
Clinical stages												
I + II	131/1244	28/200	1.34 (0.87–2.07)	0.191	131/1245	28/199	1.34 (0.87–2.08)	0.184	129/1240	30/204	1.42 (0.93–2.17)	0.107
III + IV	71/1244	18/200	1.57 (0.92–2.69)	0.101	73/1245	16/199	1.36 (0.78–2.39)	0.279	69/1240	20/204	**1.75 (1.04–2.95)**	**0.034**

The results were in bold if the 95% CI excluded 1 or *P* value less than 0.05

*AOR* adjusted odds ratio; *CI* confidence interval.

^a^Adjusted for age and gender, omitting the corresponding stratification factor.

### Haplotype analysis

We assessed whether the haplotypes of the three *METTL1* SNPs were associated with hepatoblastoma risk in the following order: rs2291617, rs10877013, and rs10877012 (Table 3). The GTT haplotype was defined as the reference group. We found a significantly elevated hepatoblastoma risk in children with the haplotype of GTG (adjusted OR = 3.87, 95% CI  1.52–9.89, *P* = 0.005), TTT (adjusted OR = 12.09, 95% CI  3.77–38.81, *P* < 0.0001), and TCT (adjusted OR = 16.95, 95% CI  3.50-81.97, *P* = 0.0004).

**Table 3:** Association between inferred haplotypes of *METTL1* gene and hepatoblastoma risk

Haplotypes ^a^	Cases (n = 616)	Controls (n = 2888)	Crude OR (95% CI)	*P*	Adjusted OR ^b^ (95% CI)	*P* ^b^
	No. (%)	No. (%)				
GTT	367 (59.58)	1816 (62.88)	1.00		1.00	
GTG	8 (1.30)	10 (0.35)	**3.96 (1.55–10.10)**	**0.004**	**3.87 (1.52–9.89)**	**0.005**
GCT	2 (0.32)	4 (0.14)	2.47 (0.45–13.56)	0.297	2.43 (0.44–13.34)	0.306
GCG	0 (0.00)	3 (0.10)	/	/	/	/
TTT	10 (1.62)	4 (0.14)	**12.37 (3.86–39.66)**	**< 0.0001**	**12.09 (3.77–38.81)**	**< 0.0001**
TTG	17 (2.76)	53 (1.84)	1.59 (0.91–2.77)	0.105	1.58 (0.91–2.77)	0.106
TCT	7 (1.14)	2 (0.07)	**17.32 (3.58–83.70)**	**0.0004**	**16.95 (3.50-81.97)**	**0.0004**
TCG	205 (33.28)	996 (34.49)	1.02 (0.84–1.23)	0.848	1.02 (0.84–1.23)	0.859

The results were in bold if the 95% CI excluded 1 or *P* value less than 0.05

*OR* odds ratio; *CI* confidence interval

^a^The haplotype order was rs2291617, rs10877013, and rs10877012

^b^Obtained in logistic regression models with adjustment for age and gender

## Discussion

Hepatoblastoma is rare but harmful, especially among high-risk populations. Children with genetic syndromes that increase the risk of hepatoblastoma are recommended to undergo screening every three months after birth. Because of the lack of reliable genetic susceptibility biomarkers, screening solely relies on whole abdominal ultrasound and AFP serum examination [26]. Susceptibility genes, environmental influences, and developmental processes are considered the most critical factors that affect pediatric cancer risk in children [27]. Similar to adult cancer, early diagnosis is a critical factor in curing many types of childhood cancers.

Case-control studies are a powerful tool for discovering disease-predisposing loci; however, such studies are incredibly uncommon in hepatoblastoma due to the scarcity of patient samples. For instance, in 48 Caucasian children with hepatoblastoma and 180 healthy controls, Pakakasama and colleagues found that a polymorphism (G to A) located in the *myeloperoxidase* (*MPO*) gene promoter was associated with decreased hepatoblastoma risk [7]. Later, the same team studied 84 children with hepatoblastoma and demonstrated that a polymorphism (G to A) at codon 242 of *CCND1*, a gene encoding cyclin D1, was associated with the age of disease onset [8]. Over the past years, we examined a pediatric cohort of 313 hepatoblastoma and 1446 controls and identified a number of loci associated with the risk of hepatoblastoma in the following genes: *LINC00673*, *NRAS*, *KRAS*, *TP53*, *HMGA2*, *miR-34b/c*, as well as DNA repair genes [9, 10, 28–30]. Posttranscriptional modifications of RNA can regulate the fate of the transcript, and therefore, proteins related to RNA modification are essential to maintain homeostasis.

We have previously shown that several RNA m6A-mediated genes (i.e., *METTL3*, *METTL14*, *WTAP*, *YTHD1*, *YTHDC1*, and *ALKBH5*) are associated with hepatoblastoma susceptibility [11, 12, 22, 31–33]. *YTHDF1* gene rs6090311 was associated with decreased hepatoblastoma risk [11], whereas rs7766006 in the *WTAP* gene was associated with an increased risk of hepatoblastoma [22]. The m7G modification also plays an important role in the fate of RNA. However, the contributions of genetic variants in m7G-mediated genes to disease susceptibility have rarely been investigated. To date, only one study indicated the possible association of *METTL1* gene polymorphisms with the risk of disorders. A GWAS conducted by the Australian and New Zealand Multiple Sclerosis Genetics Consortium discovered that rs703842 positioned at the 3′ untranslated region (3′ UTR) of the *METTL1* gene was associated with the risk of multiple sclerosis [34]. This study examined the additive effect of three *METTL1* genetic polymorphisms on hepatoblastoma susceptibility, although none of these SNPs showed significant effects as a single locus.

METTL1 forms a heterodimeric protein complex with WD repeat domain 4 (WDR4) to regulate the expression, localization, and function of mRNA, miRNA, tRNA, and rRNA by catalyzing m7G modifications [16, 17, 35]. The roles of METTL1 in oncogenesis remain controversial. Some studies suggest METTL1 is a potential tumor suppressor. Liu et al. demonstrated that forced expression of METTL1 sensitized colon cancer cells to cisplatin by activating the miR-149-3p/S100A4/p53 axis [20]. Studies show that many tumor suppressor microRNAs harbor 7-methylguanosine (m7G), including *let-7e* [21]. Pandolfini et al. indicated that METTL1 is responsible for promoting m7G modifications on miRNAs. METTL1 accelerated let-7 miRNA processing, thereby inhibiting lung cancer cell migration. Mechanistically, m7G modification in primary miRNA (pri-miRNA) transcripts prevents the formation of inhibitory RNA secondary structures and therefore facilitates the maturation of miRNAs [21]. In contrast, the tumor-promoting role of METTL1 was reported in hepatocellular carcinoma (HCC), lung cancer, and head and neck squamous cell carcinoma [15–18]. Tian et al. observed that METTL1 upregulation is associated with poor prognosis in HCC. METTL1 promoted HCC by inhibiting PTEN and activating the AKT signaling pathway. In addition, the Orellana group provided evidence of METTL1’s oncogenic role [15]. Analysis of TCGA datasets revealed that METTL1 is frequently amplified and overexpressed in various human cancers, such as glioblastoma and sarcoma [15]. Functional experiments confirmed that METTL1 overexpression led to oncogenic cell transformation, but silencing METTL1 reduced the abundance of m7G-modified tRNAs and inhibited oncogenicity [15]. Primarily, METTL1-mediated m7G modification of Arg-TCT tRNA increased the expression levels of cell cycle regulators, thereby inducing oncogenic transformation [15]. Taken together, the role of METTL1 in tumorigenesis differs based on the context. In the present study, we identified METTL1 as a gene associated with hepatoblastoma susceptibility. Although the implications of METTL1 in hepatoblastoma carcinogenesis have not been reported, two new publications showed that METTL1 is related to radiotherapy resistance [36] and recurrence post-radiofrequency ablation [37] in hepatocellular carcinoma. In vitro and in vivo studies should be performed to demonstrate the involvement of METTL1 in hepatoblastoma development and/or progression in the future.

The weak effects of single SNPs on disease risk have greatly limited their clinical translation. Encouragingly, accumulating evidence has indicated that risk scores derived from a panel of disease-causing SNPs are promising markers [38, 39]. Cuzick et al. demonstrated that the SNP88 risk score was predictive of breast cancer risk and substantially improved risk predictive accuracy when combined with the existing breast cancer risk assessment tool Tyrer-Cuzick (TC) [38]. In addition, the Whitfield group reported that a genetic risk score based on 3 SNPs and diabetes status could robustly discriminate cirrhosis risk [39]. Therefore, it is essential to identify more hepatoblastoma susceptibility to develop risk prediction panels of polygenic SNPs.

Even though this study included a relatively large cohort with samples collected from seven independent participating hospitals across China, the limitations should be discussed. First, hepatoblastoma is a complex disease that is likely driven by many genetic factors, environmental factors, and gene-environment interactions. Here, we only considered potential functional genetic variants while ignoring environmental factors, such as parental tobacco consumption and prematurity. Further attempts should be made to collect information on the other confounding factors that may influence the outcome of genotyping variants. Second, functional experiments need to be performed to validate the connection between variant genotypes and phenotypes. Third, the number of cases should be expanded further to increase the statistical power. Finally, the major weakness of the study is the lack of biologically relevant evidence that METTL1 plays a role in hepatoblastoma pathogenesis.

Overall, we demonstrated that three SNPs in the *METTL1* gene synergistically confer increased hepatoblastoma susceptibility. Replication studies should be carried out to validate our findings prior to clinical translation.

## Supplementary Information

Below is the link to the electronic supplementary material.

**Additional file 1: Table S1.** Frequency distribution of selected variables in hepatoblastoma patients and cancer-free controls.

## Data Availability

The original contributions presented in the study are included in the article/Supplementary Material; further inquiries can be directed to the corresponding authors.

## References

[1] Spector LG, Birch J (2012). The epidemiology of hepatoblastoma. Pediatr Blood Cancer.

[2] Rougemont AL, McLin VA, Toso C, Wildhaber BE (2012). Adult hepatoblastoma: learning from children. J Hepatol.

[3] Czauderna P, Lopez-Terrada D, Hiyama E, Haberle B, Malogolowkin MH, Meyers RL (2014). Hepatoblastoma state of the art: pathology, genetics, risk stratification, and chemotherapy. Curr Opin Pediatr.

[4] Marin JJG, Cives-Losada C, Asensio M, Lozano E, Briz O, Macias RIR (2019). Mechanisms of anticancer drug resistance in hepatoblastoma. Cancers (Basel).

[5] Meyers RL, Maibach R, Hiyama E, Häberle B, Krailo M, Rangaswami A (2017). Risk-stratified staging in paediatric hepatoblastoma: a unified analysis from the Children’s Hepatic tumors International Collaboration. Lancet Oncol.

[6] Sorahan T, Lancashire RJ (2004). Parental cigarette smoking and childhood risks of hepatoblastoma: OSCC data. Br J Cancer.

[7] Pakakasama S, Chen TT, Frawley W, Muller C, Douglass EC, Tomlinson GE (2003). Myeloperoxidase promotor polymorphism and risk of hepatoblastoma. Int J Cancer.

[8] Pakakasama S, Chen TT, Frawley W, Muller CY, Douglass EC, Lee R (2004). CCND1 polymorphism and age of onset of hepatoblastoma. Oncogene.

[9] Li L, Zhuo Z, Yang Z, Zhu J, He X, Yang Z (2020). HMGA2 polymorphisms and hepatoblastoma susceptibility: a five-center case-control study. Pharmgenomics Pers Med.

[10] Liu P, Zhuo ZJ, Zhu J, Yang Z, Xin Y, Li S (2020). Association of TP53 rs1042522 C > G and miR-34b/c rs4938723 T > C polymorphisms with hepatoblastoma susceptibility: a seven-center case-control study. J Gene Med.

[11] Luo Z, Li G, Wang M, Zhu J, Yang Z, Li Y (2020). YTHDF1 rs6090311 A > G polymorphism reduces hepatoblastoma risk: evidence from a seven-center case-control study. J Cancer.

[12] Chen H, Duan F, Wang M, Zhu J, Zhang J, Cheng J (2021). Polymorphisms in METTL3 gene and hepatoblastoma risk in Chinese children: a seven-center case-control study. Gene.

[13] Cowling VH (2009). Regulation of mRNA cap methylation. Biochem J.

[14] Zhang LS, Liu C, Ma H, Dai Q, Sun HL, Luo G (2019). Transcriptome-wide mapping of internal N(7)-methylguanosine methylome in mammalian mRNA. Mol Cell.

[15] Orellana EA, Liu Q, Yankova E, Pirouz M, De Braekeleer E, Zhang W (2021). METTL1-mediated m(7)G modification of Arg-TCT tRNA drives oncogenic transformation. Mol Cell.

[16] Chen J, Li K, Chen J, Wang X, Ling R, Cheng M (2022). Aberrant translation regulated by METTL1/WDR4-mediated tRNA N7-methylguanosine modification drives head and neck squamous cell carcinoma progression. Cancer Commun (Lond).

[17] Ma J, Han H, Huang Y, Yang C, Zheng S, Cai T (2021). METTL1/WDR4-mediated m(7)G tRNA modifications and m(7)G codon usage promote mRNA translation and lung cancer progression. Mol Ther.

[18] Tian QH, Zhang MF, Zeng JS, Luo RG, Wen Y, Chen J (2019). METTL1 overexpression is correlated with poor prognosis and promotes hepatocellular carcinoma via PTEN. J Mol Med (Berl).

[19] Chen Z, Zhu W, Zhu S, Sun K, Liao J, Liu H (2021). METTL1 promotes hepatocarcinogenesis via m(7) G tRNA modification-dependent translation control. Clin Transl Med.

[20] Liu Y, Yang C, Zhao Y, Chi Q, Wang Z, Sun B (2019). Overexpressed methyltransferase-like 1 (METTL1) increased chemosensitivity of colon cancer cells to cisplatin by regulating miR-149-3p/S100A4/p53 axis. Aging.

[21] Pandolfini L, Barbieri I, Bannister AJ, Hendrick A, Andrews B, Webster N (2019). METTL1 promotes let-7 MicroRNA processing via m7G methylation. Mol Cell.

[22] Zhuo ZJ, Hua RX, Chen Z, Zhu J, Wang M, Yang Z (2020). WTAP gene variants confer hepatoblastoma susceptibility: a seven-center case-control study. Mol Ther Oncolytics.

[23] Roebuck DJ, Aronson D, Clapuyt P, Czauderna P, de Ville de Goyet J, Gauthier F (2007). 2005 PRETEXT: a revised staging system for primary malignant liver tumours of childhood developed by the SIOPEL group. Pediatr Radiol.

[24] Bian J, Zhuo Z, Zhu J, Yang Z, Jiao Z, Li Y (2020). Association between METTL3 gene polymorphisms and neuroblastoma susceptibility: a nine-centre case-control study. J Cell Mol Med.

[25] Zhang Z, Zhang R, Zhu J, Wang F, Yang T, Zou Y (2017). Common variations within HACE1 gene and neuroblastoma susceptibility in a Southern Chinese population. Onco Targets Ther.

[26] Kalish JM, Doros L, Helman LJ, Hennekam RC, Kuiper RP, Maas SM (2017). Surveillance recommendations for children with overgrowth syndromes and predisposition to Wilms tumors and hepatoblastoma. Clin Cancer Res.

[27] Pakakasama S, Tomlinson GE (2002). Genetic predisposition and screening in pediatric cancer. Pediatr Clin North Am.

[28] Zhuo Z, Lin A, Zhang J, Chen H, Li Y, Yang Z (2021). Genetic variations in base excision repair pathway genes and risk of hepatoblastoma: a seven-center case-control study. Am J Cancer Res.

[29] Zhuo Z, Miao L, Hua W, Chen H, Yang Z, Li Y (2021). Genetic variations in nucleotide excision repair pathway genes and hepatoblastoma susceptibility. Int J Cancer.

[30] Yang T, Wen Y, Li J, Tan T, Yang J, Pan J (2019). NRAS and KRAS polymorphisms are not associated with hepatoblastoma susceptibility in Chinese children. Exp Hematol Oncol.

[31] Chen H, Chen Z, Wang M, Zhang J, Li Y, Li L (2022). METTL14 gene polymorphisms influence hepatoblastoma predisposition in Chinese children: Evidences from a seven-center case-control study. Gene.

[32] Ren H, Zhuo ZJ, Duan F, Li Y, Yang Z, Zhang J (2021). ALKBH5 gene polymorphisms and hepatoblastoma susceptibility in Chinese children. J Oncol.

[33] Chen H, Li Y, Li L, Zhu J, Yang Z, Zhang J (2020). YTHDC1 gene polymorphisms and hepatoblastoma susceptibility in Chinese children: a seven-center case-control study. J Gene Med.

[34] Australia, New Zealand Multiple Sclerosis Genetics C (2009). Genome-wide association study identifies new multiple sclerosis susceptibility loci on chromosomes 12 and 20. Nat Genet.

[35] Lin S, Liu Q, Lelyveld VS, Choe J, Szostak JW, Gregory RI (2018). Mettl1/Wdr4-mediated m(7)G tRNA methylome is required for normal mRNA translation and embryonic stem cell self-renewal and differentiation. Mol Cell.

[36] Liao J, Yi Y, Yue X, Wu X, Zhu M, Chen Y (2022). METTL1 is required for non-homologous end joining repair and renders hepatocellular carcinoma resistant to radiotherapy. Hepatology.

[37] Zeng X, Liao G, Li S, Liu H, Zhao X, Li S (2022). Eliminating METTL1-mediated accumulation of PMN-MDSCs prevents HCC recurrence after radiofrequency ablation. Hepatology.

[38] Cuzick J, Brentnall AR, Segal C, Byers H, Reuter C, Detre S (2017). Impact of a panel of 88 single nucleotide polymorphisms on the risk of breast cancer in high-risk women: results from two randomized tamoxifen prevention trials. J Clin Oncol.

[39] Whitfield JB, Schwantes-An TH, Darlay R, Aithal GP, Atkinson SR, Bataller R (2022). A genetic risk score and diabetes predict development of alcohol-related cirrhosis in drinkers. J Hepatol.

