# Supplementary material for: METTL1 gene polymorphisms synergistically confer hepatoblastoma susceptibility
Source: Discov Oncol. 2022 Aug 20;13:77. doi: 10.1007/s12672-022-00545-7 (PMC9392666; doi:10.1007/s12672-022-00545-7)
Supplement: Supplementary file 1 — Additional file 1: Table S1. Frequency distribution of selected variables in hepatoblastoma patients and cancer-free controls. [file 12672_2022_545_MOESM1_ESM.doc]

| **Table S1**. Frequency distribution of selected variables in hepatoblastoma patients and cancer-free controls | | | | | |
| --- | --- | --- | --- | --- | --- |
| Variables | Cases (n=313) | | Controls (n=1446) | | *P* ^a^ |
|  | No. | % | No. | % |  |
| Age |  |  |  |  | 0.251 ^b^ |
| <17 months | 168 | 53.67 | 642 | 44.40 |  |
| ≥17 months | 145 | 46.33 | 804 | 55.60 |  |
| Gender |  |  |  |  | 0.983 |
| Female | 129 | 41.21 | 595 | 41.15 |  |
| Male | 184 | 58.79 | 851 | 58.85 |  |
| Clinical stages |  |  |  |  |  |
| I | 97 | 30.99 | / | / |  |
| II | 63 | 20.13 | / | / |  |
| III | 64 | 20.45 | / | / |  |
| IV | 27 | 8.63 | / | / |  |
| NA | 62 | 19.81 | / | / |  |
| SD, standard deviation, NA, not available.  ^a^ Two-sided χ^2^ test for distributions between hepatoblastoma cases and cancer-free controls.  ^b^ T-test for age distribution between hepatoblastoma patients and cancer-free controls. | | | | | |
